# Computational analysis of dimer G6PD structure to elucidate pathogenicity of G6PD variants

**DOI:** 10.37796/2211-8039.1431

**Published:** 2024-03-01

**Authors:** Shamini Chandran, Naveen Eugene Louis, Syazwani Itri Amran, Nurriza Ab Latif, Muaawia Ahmed Hamza, Mona Alonazi

**Affiliations:** a Universiti Teknologi Malaysia, Johor, Malaysia; b King Fahad Medical City, Riyadh, Kingdom of Saudi Arabia; c King Saud University, Riyadh, Kingdom of Saudi Arabia

**Keywords:** G6PD deficiency, Molecular dynamic simulation (MDS), G6PD dimer, Trajectory analysis

## Abstract

An inherent genetic enzyme disorder in humans, known as glucose-6-phosphate dehydrogenase (G6PD) deficiency, arises due to specific mutations. While the prevailing approach for investigating G6PD variants involves biochemical analysis, the intricate structural details remain limited, impeding a comprehensive understanding of how different G6PD variants of varying classes impact their functionality. This study 22 examined the dynamic properties of G6PD wild types and six G6PD variants from 23 different classes using molecular dynamic simulation (MDS). The wild-type and variant 24 G6PD structures unveil high fluctuations within the amino acid range of 274–515, the structural NADP^+^ binding site, pivotal for enzyme dimerization. Specifically, two variants, G6PD_Zacatecas_ (R257L) and G6PD_Durham_ (K238R), demonstrate compromised structural stability at the dimer interface, attributable to the disruption of a salt bridge involving Glu 206 and Lys 407, along with the disturbance of hydrogen bonds formed by Asp 421 at the βN-βN sheets. Consequently, this impairment cascades to affect the binding affinity of crucial interactions, such as Lys 171-Glucose-6-Phosphate (G6P) and Lys 171-catalytic NADP^+^, leading to diminished enzyme activity. This study underscores the utility of computational in silico techniques in predicting the structural alterations and flexibility of G6PD variants. This insight holds promise for guiding future endeavors in drug development targeted at mitigating the impacts of G6PD deficiency.

## 1. Introduction

G6PD enzyme (E.C.1.1.1.49) plays a crucial role in the pentose phosphate pathway, which protects the red blood cells by maintaining NADPH levels against oxidative damage. G6PD deficiency causes hemolysis-prone red blood cells, resulting in a wide range of medical consequences [1,2]. Patients with G6PD deficiency often suffer from acute hemolytic anemia, chronic non-spherocytic hemolytic anemia, favism, and neonatal jaundice [38]. Additionally, it increases the likelihood of developing diabetes mellitus, hypertension, cancer, and cataracts [3]. Clinical attributes are significantly more dominant in males, as they possess only one Xchromosome. Conversely, females who are heterozygous with one normal G6PD gene and one mutated G6PD gene on the other chromosome can undergo a 50 % deficiency effect [4]. World Health Organization (WHO) classified G6PD variants into four classes based on median enzyme activity and hemolysis severity. Class A G6PD variants cause Chronic NonSpherocytic Hemolytic Anemia (CNSHA) with less than 20 % median G6PD activity. Class B has median G6PD activity of less than 45 % and causes acute hemolysis, while class C indicates no hemolysis with median G6PD activity between 60 % and 150 %. Finally, class U G6PD variants have uncertain clinical significance. According to WHO data, over 230 G6PD variants have been discovered in the past 36 years.

**Fig. 1:**
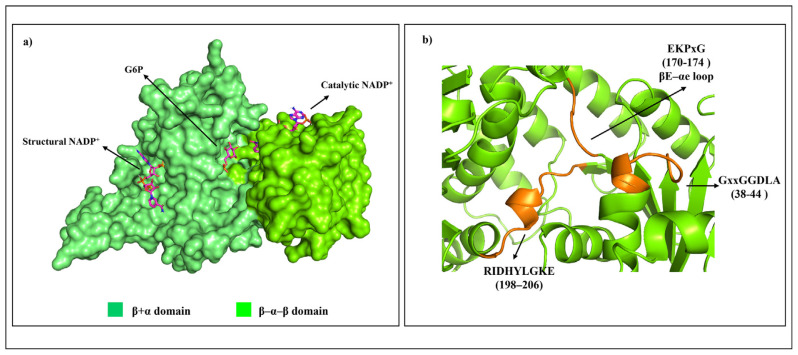
a) Human native G6PD enzyme structure, showing the ligand (sticks) in the monomer. b) Location of conserved amino acids sequence (orange cartoon) in the monomer G6PD enzyme based on multiple sequence alignment. The figure was prepared using PyMOL software and adapted from PDB entries 2BHL and 2BH9.

**Fig. 2:**
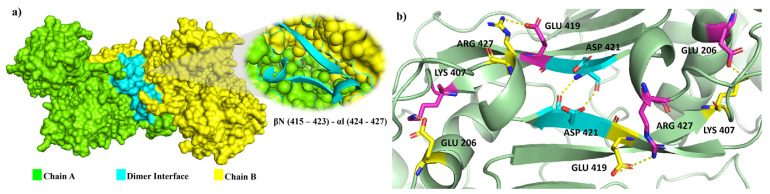
a) Visual representation of G6PD dimer structure with dimer interface βN (415–423) - αl (424–427). b) Human native G6PD enzyme’s crystallographic structure shows four salt bridges forming amino acid residues (pink stick represents chain A; yellow stick represents chain B) at the dimerization site. The Cyan strand represent conserved region Leu 420, Asp 421, and Leu 422 with stick representation of Asp 421-Asp 421 hydrogen bonds at the dimer interface. The figure was prepared using PyMOL software (Adapted from PDB entries 2BHL and 2BH9).

The active human G6PD enzyme is composed of tetramer and dimer forms [5]. Each monomer has a substrate-binding site and two NADP^+^ coenzyme binding sites. There are two domains in each monomeric structure: the β–α–β domain (N terminal) and β+α domain (C terminal). The β–α–β domain includes amino acids from 1 to 200 with a catalytic NADP^+^ binding site shown in Fig. 1(a), while the β+α domain consists of amino acids from 201 to 515 with a Glucose-6-Phosphate (G6P) substrate binding site and a structural NADP^+^ binding site represented by an antiparallel nine-strand sheet [6]. The β + α domains at βN (415–423) - αl (424–427) form the extensive dimer relation shown in Fig. 2(a). Leu 420, Asp 421, and Leu 422 located at βN are highly conserved in all species, indicating their importance for enzyme stability and activity. Structural NADP^+^ is crucial for maintaining dimer structure in higher organisms [3]. These ligands influence the formation of active enzymes either in the form of dimer or tetramer [7]. A high concentration of G6P can disrupt dimer formation, whereas the presence of NADP^+^ favors tetramer formation [8].

The dimer interface is stabilized by four salt bridges connecting Glu 206 to Lys 407 and Glu 419 to Arg 427, as indicated in Fig. 2(b), with only Lys 407 being wholly conserved.

The hydrogen bond between Asp 421-Asp 421 that connects the two βN strands of the β sheets is crucial for structural stability [9]. Three conserved sequences have been identified as shown in Fig. 1(b): RIDHYLGKE, a nine-residue peptide at amino acid at position 198–206 [10], conserved Lys 205, which is critical for substrate binding and catalysis of the enzyme. The next GxxGGDLA at position 38–44 connects the catalytic NADP^+^ binding site. The final conserved sequence is EKPxG located at amino acid 170–174, where Lys 171 is required to properly position the G6P substrate and NADP^+^ coenzymes during the enzymatic process [10].

Published biochemical data reported that the catalytic efficiency and protein instability contribute to the clinical phenotypes in G6PD variants [11,12]. Simulations have been carried out in the past on both the monomeric and dimeric forms of G6PD [13]. However, the mechanism of mutation-induced structural stability and the physiological influence of the ligands in the G6PD enzyme structure remains unclear to date. Hence, this study focuses on constructing a complete structure of the G6PD dimer mutant by using in silico site-directed mutagenesis. Then, characterizes the structural changes through molecular dynamic simulation (MDS) analysis. The GROMACS program utilizes the structural model of G6PD wild type and variants bound with G6P substrate and NADP^+^ cofactors [14,15]. Protein backbone Root Mean Square Deviation (RMSD), Carbon Alpha Root Mean Square Fluctuation (RMSF), Radius of Gyration (Rg), Solvent Accessibility Surface Area (SASA), intermolecular hydrogen bond of enzyme, and confirmational changes derived from MDS were used to analyze structural stability and flexibility. A total of six variants which includes G6PD_Zacatecas_ (R257L), G6PD_Durham_ (K238R), G6PD_Valladolid_ (R136C), G6PD_Mediterranean_ (S188F), G6PD_A+_ (N126D) and G6PD_Mexico City_ (R227Q) were analyzed in this study.

## 2. Materials and methodology

### 2.1. Variant selection

An extensive search was conducted in databases such as Science Direct and Web of Science using the terms “G6PD deficit”, “Glucose-6-phosphate deficiency,” and “G6PDD”. Variants were chosen based on criteria such as being published in primary research journals and having reported biochemical data. The background of the selected predominant variants was studied, and mutation points were determined. G6PD_Zacatecas_ (R257L), G6PD_Durham_ (K238R), G6PD_Valladolid_ (R136C), G6PD_Mediterranean_ (S188F), G6PD_A+_ (N126D) and G6PD_Mexico City_ (R227Q) were selected for this study [16–22].

### 2.2. Site-directed mutagenesis

The structure of the human G6PD dimer enzyme with the ligands was prepared using Autodock software using two crystal structures from the PDB database (PDB entries 2BH9 and 2BHL) in the previous study. PyMOL software performed in silico site-directed mutagenesis on a complex G6PD enzyme dimer structure [23]. The resulting structures were saved in PDB format and validated using the SPDB viewer tool. The mutated dimer structures were then loaded into the SPDB viewer to confirm the location of the mutated residue [24].

### 2.3. Molecular dynamic simulation (MDS)

The G6PD dimer structures were simulated for 100ns using GROMACS 4.6.7 package [25]. Ligands were separated from the protein and saved as pdb files. Topologies for ligands and proteins were constructed using different servers. The GRO-MOS96 53a7 force field was used to build the topologies for the proteins via the pdb2gmx program. The ligand topologies were generated using the Automated Topology Builder (ATB) server [26]. The ligand pdb and its files were saved in the directory to reconstruct the protein-ligand complex.

The protein, ligands, and minimized system were combined to create a complex structure.gro file. The complex protein structure was solvated in a cubic box with the SPC water model. To neutralize electric charges for simulation complexes, water molecules were replaced by Na^+^ and Cl^−^ ions within the box [27]. The system’s energy was minimized, and output was checked using xmgrace software. The neutralized method was equilibrated for 50,000 steps using the NVT and NPT systems [27]. Following this, a production MD stage was performed for 100 ns at a constant temperature of 300 K and pressure of 1.0 atm.

### 2.4. Analysis

The root mean square deviation (RMSD), root mean square fluctuation (RMSF), Radius of gyration (Rg), Solvent accessibility surface area(SASA), intermolecular hydrogen bonds, and post structures were calculated using gmx rms, gmx rmsf, gmx gyration,gmx sasa, gmx hbonds and gmx trjconv, respectively [28,29]. The results are graphically displayed via.

QTGRACE software. The structural configuration was analyzed using PyMOL software.

## 3. Results and discussion

### 3.1. Mutation site

**Fig. 3:**
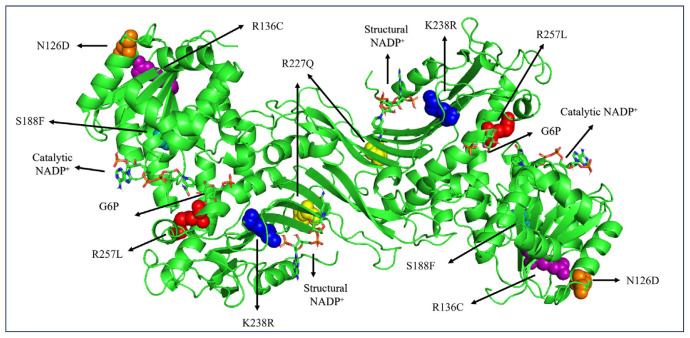
The complete dimeric structure of G6PD (PDB entries 2BHL and 2BH9) is shown with structural NADP^+^, catalytic NADP^+^, and the G6P substrate in cartoon and stick representation. Location of G6PD_Zacatecas_ (red spheres), G6PD_Durham_ (blue spheres), G6PD_Valladolid_ (purple spheres), G6PD_Mediterranean_ (teal spheres), G6PD_MexicoCity_ (yellow spheres), and G6PD_A+_ (orange spheres) showed respectively.

In this study, six G6PD variants were chosen from different G6PD classes namely G6PD_Zacatecas_ (R257L), G6PD_Durham_ (K238R), G6PD_Valladolid_ (R136C), G6PD_Mediterranean_ (S188F), G6PD_A+_ (N126D) and G6PD_Mexico City_ (R227Q) as shown in Fig. 3. All the variants have a noticeable single missense mutation which is prominent in different regions of the world, such as Europe (G6PD_Valladolid_ and G6PD_Mediterranean_), Africa (G6PD_A+_), and America (G6PD_Zacatecas_, G6PD_Durham_, and G6PD_Mexico City_) [30].

Analysis of the structural changes resulting from each missense mutation were conducted by calculating the average distance and number of hydrogen bonds connecting the mutation site with its adjacent residues.

**Fig. 4:**
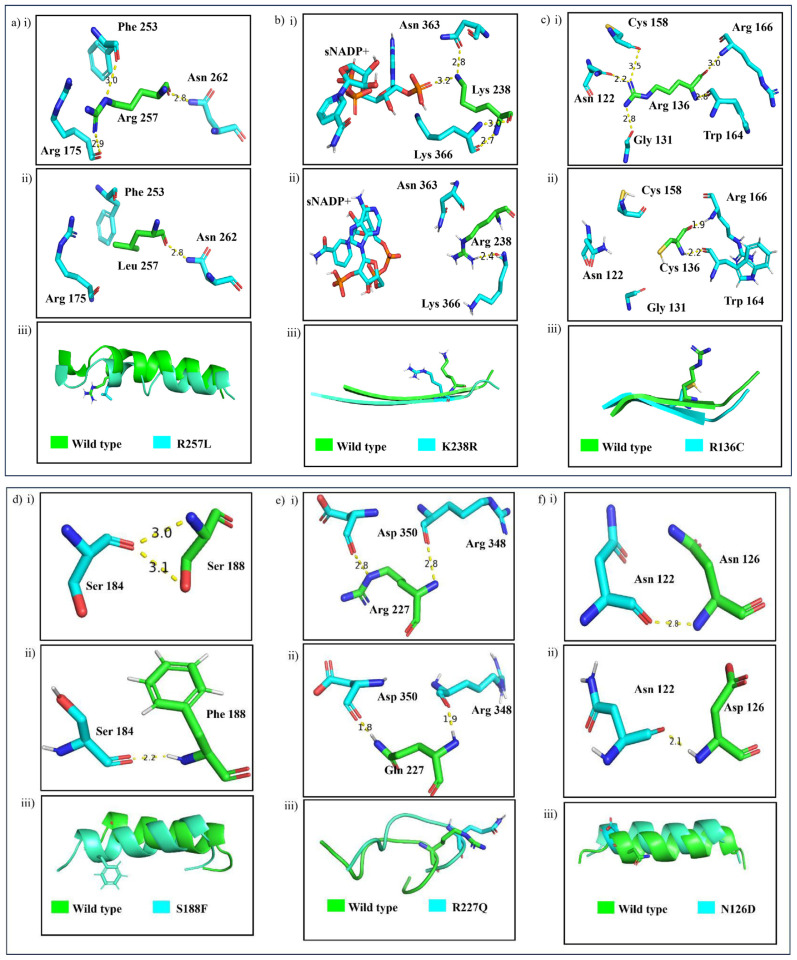
The intermolecular interactions in dimer i) wild type, ii) variant structures and iii) superimpose structure at mutation site of a) G6PD_Zacatecas_ (R257L), b) G6PD_Durham_ (K238R), c) G6PD_Valladolid_ (R136C), d) G6PD_Mediterranean_ (S188F), e) G6PD_MexicoCity_ (R227Q), and f) G6PD_A+_ (N126D).

As shown in Fig. 3, the G6PD_Zacatecas_ (R257L) mutation causes a change from polar Arg to nonpolar Leu at amino acid position 257 in a helical structure close to the G6P substrate at the αi loop. The mutation of Arg to Leu led to a loss of hydrogen bonds between the side chains Fig. 4 (a). The mutation also induces loss of salt bridge interactions between Lys 257 and Glu 473 in tetrameric form. Replacement of non-polar Leu exposes a hidden hydrophobic region on the enzyme, presumably resulting in loss of secondary structure [1]. Mutation in G6PD_Durham_ (K238R) demonstrated the replacement of polar Lys to the positive charged Arg residue at amino acid 238. This mutation led to a decrease in the distance by a factor of 0.6 Å and retained only one hydrogen bond between α-βG and βK at the dimer interface domain (refer Table 1). The G6PD_Durham_ (K238R) mutation unequivocally diminishes the affinity of structural NADP^+^. This is apparent from the absence of a crucial hydrogen bond between highly conserved amino acid 238 and structural NADP^+^ which is clearly illustrated in Fig. 4 (b).

G6PD_Valladolid_ (R136C) represents substitution between two polar amino acids, Arg to Cys, at amino acid 136. The mutation is located at the highly conserved βD strand near catalytic NADP^+^. This mutation retained two bonds from the wild type: Trp 164 and Arg 166 at αd-βE loop, with bond lengths of 2.2 Å and 1.9 Å, respectively. In contrast, a mutation in G6PD_Mediterranean_ (S188F) showed a change of polar Ser to non-polar Phe on αe strand at amino acid 188. It is important to note that even though the mutation is not situated in the conserved region, any alteration in polarity could potentially interfere with the formation of the enzyme’s secondary structure. In the wild type, Ser 188 makes two hydrogen bonds with Ser 184 in the same αe strand. However, substituting Phe causes the loss of a hydrogen bond and disrupts the formation of the secondary structure of the enzyme.

In G6PD_MexicoCity_ (R227Q), polar Arg at position 227 changed to polar Gln at αg-βG loop and retained the two hydrogen bonds between Arg 348 and Asp 350 with reduced distance from wild type. The substitution of Gln leads to steric hindrance, probably affecting the structural conformation [31]. It has been reported by G6PD_A+_ (N126D) that a negatively charged Asp residue situated at position 126 (ac strand) has been replaced with a polar Asn residue. This variant has resulted in the reduction of the distance of the ac strand, thereby allowing it to retain a hydrogen bond with Asn 122.

G6PD_Zacatecas_, G6PD_Durham_, G6PD_Valladolid_, and G6PD_Mediterranean_ exhibited reduced affinity based on the presence and proximity of intermolecular interactions with adjacent residues. In contrast, G6PD_MexicoCity_ and G6PD_A+_ displayed comparable affinity.

### 3.2. Trajectory analysis

Molecular Dynamic Simulation was performed for dimer wild type and six G6PD variants from different classes (see Table 1). The Gromos54a7 forcefield in the GROMACS package was used to simulate the structures for 100ns. The analysis of molecular dynamic simulation trajectories yielded the mean values for RMSD, RMSF, Rg, and SASA, which are presented in Table 2.

**Fig. 5:**
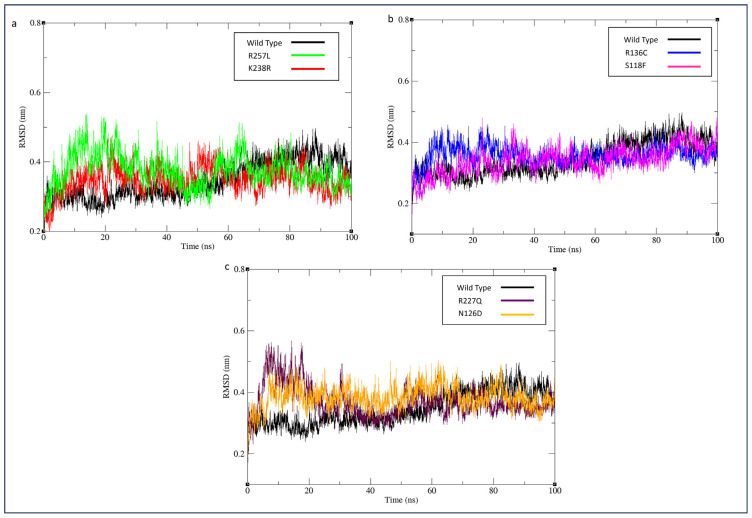
MD simulation of wild type against variants. RMSD of the backbone conformation was shown as a function of simulation time (100 ns) at 300 K. RMSD plotted as (a) G6PD_Zacatecas_ (R257L) and G6PD_Durham_ (K238R), (b) G6PD_Valladolid_ (R136C) and G6PD_Mediterranean_ (S188F), and c) G6PD_MexicoCity_ (R227Q), and G6PD_A+_ (N126D).

To assess the Cα backbone divergence between the variant protein structure and the wild type G6PD enzyme, the RMSD value was employed. A higher RMSD value unequivocally indicates lower protein structural stability throughout the simulation. Wild type G6PD stabilized after 60ns with an average RMSD value of 0.35 nm. All the average RMSD values of variants showed the least deviation compared to the wild type. However, G6PD_Zacatecas_ (R257L), G6PD_Durham_ (K238R), and G6PD_Mediterranean_ (S188F) were observed to have unstable fluctuations throughout the simulation, as indicated in Fig. 5(a) and b). While G6PD_Valladolid_ (R136C), G6PD_A+_ (N126D) and G6PD_Mexico City_ (R227Q) started to stabilize after 60ns.

**Fig. 6:**
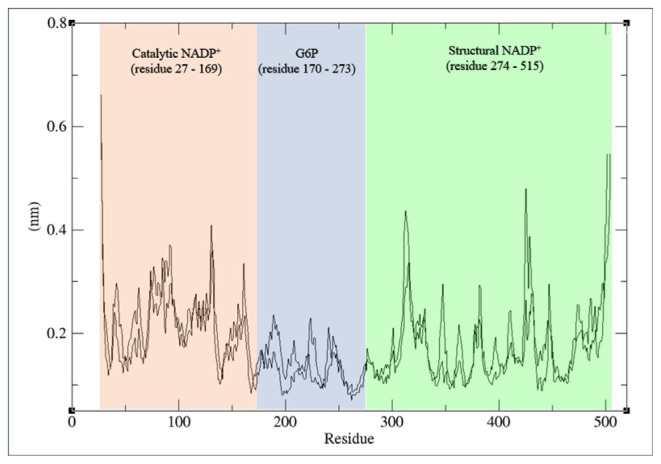
RMS Fluctuation. The root mean square fluctuation (RMSF) of the Cα backbone of wild type G6PD at 300K at 100ns.

The RMSF values of variants Cα backbone amino acid residues were computed and compared to the wild type structure. Any RMSF readings exceeding 0.05 nm (0.5 Å) from the wild type signify a substantial elevation in amino acid flexibility [19]. A greater degree of flexibility is indicated by a higher RMSF value, while a low RMSF value suggests restricted movement compared to its average position throughout the simulation [32,33]. The amino acid residues at the catalytic NADP^+^ binding site are represented by residue 27–169, the G6P substrate binding site composed of residues from position 170–273, while the structural NADP^+^ binding site is from residue 274–515, as indicated in Fig. 6. The average RMSF value of variant structures shows no drastic deviation. However, wild type and variants fluctuate more at the structural NADP^+^ site consisting of coils and β-sheets than at catalytic NADP^+^ and G6P substrate sites. The impact of intra-molecular hydrogen bonds on protein structure and dynamics is significant [34]. Importantly, intermolecular hydrogen bonds have the potential to decrease protein fluctuations and enhance rigidity. From the intramolecular hydrogen bonds results G6PD_A+_ have higher hydrogen bond with lower RMSF fluctuation.

**Fig. 7:**
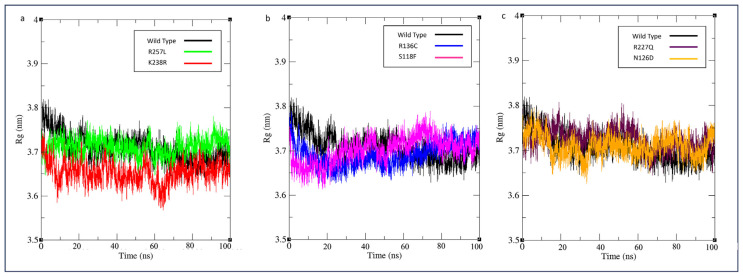
MD simulation of wild type against variants. The radius of gyration (Rg) was shown as a function of simulation time (100 ns) at 300 K. R_g_ plotted as a) G6PD_Zacatecas_ (R257L) and G6PD_Durham_ (K238R), (b) G6PD_Valladolid_ (R136C) and G6PD_Mediterranean_ (S188F), and c) G6PD_MexicoCity_ (R227Q), and G6PD_A+_ (N126D).

Determining the structural compactness of a protein is dependent on the average Rg value [35]. This value is known to decrease as the compactness of the protein increases, providing clear and indisputable evidence of its structural integrity. G6PD_Mexico City_ (R227Q) showed a slight increase in the Rg value, indicating the mutation could affect the enzyme structure’s compactness, as shown in Fig. 7. There was no significant deviation in the structural compactness among other variants, with an average Rg value ranging from 0.70 nm to 0.71 nm.

**Fig. 8:**
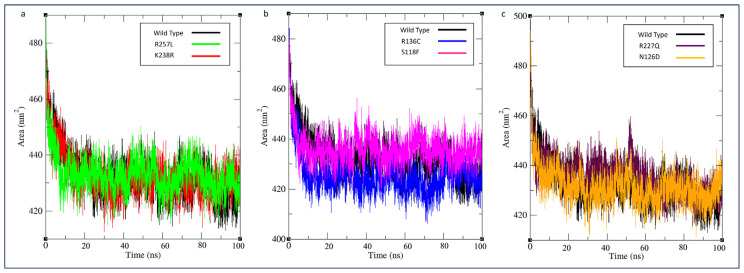
MD simulation of wild type against variants. The solvent accessibility surface (SASA) was shown as function of simulation time (100 ns) at 300 K. SASA plotted as a) G6PD_Zacatecas_ (R257L) and G6PD_Durham_ (K238R), (b) G6PD_Valladolid_ (R136C) and G6PD_Mediterranean_ (S188F), and c) G6PD_MexicoCity_ (R227Q), and G6PD_A+_ (N126D).

**Fig. 9:**
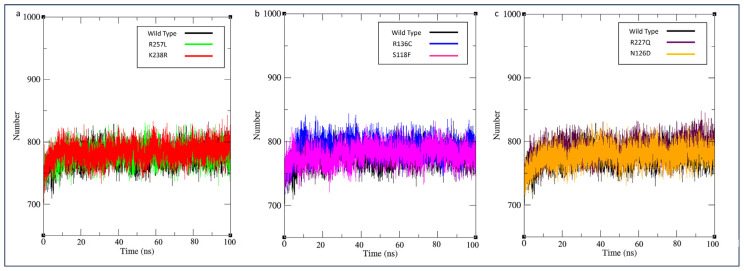
MD simulation of wild type against variants. A number of intermolecular hydrogen bonds were shown as function of simulation time (100 ns) at 300 K. Hydrogen bonds plotted as a) G6PD_Zacatecas_ (R257L) and G6PD_Durham_ (K238R), (b) G6PD_Valladolid_ (R136C) and G6PD_Mediterranean_ (S188F), and c) G6PD_MexicoCity_ (R227Q), and G6PD_A+_ (N126D).

The analysis of the accessible surface area for solvent molecules is performed using SASA results. A higher SASA value indicates a more exposed region for the solvent [36]. Variant G6PD_Mexico City_ (R227Q) showed a higher SASA value than the wild type, which denotes relative expansion of the structure, as indicated in Fig. 8. The SASA was lowered due to increased hydrophobic region exposure caused by the cysteine mutation in G6PD_Valladolid_. Moreover, it is evident from Fig. 9 that the intermolecular hydrogen bond in this specific variant has significantly increased. This is because the frequency of hydrogen-bonded side chains in proteins is inversely related to the solvent accessibility of their donors and acceptors. Thus, intramolecular hydrogen bonding favors buried and partially buried residues [37]. The SASA value and intermolecular hydrogen bond for other variants fall within the range of 432 nm^2^–436 nm^2^ and 777 to 787 bonds, respectively, with no noticeable alterations.

### 3.3. Dimer interface

**Fig. 10:**
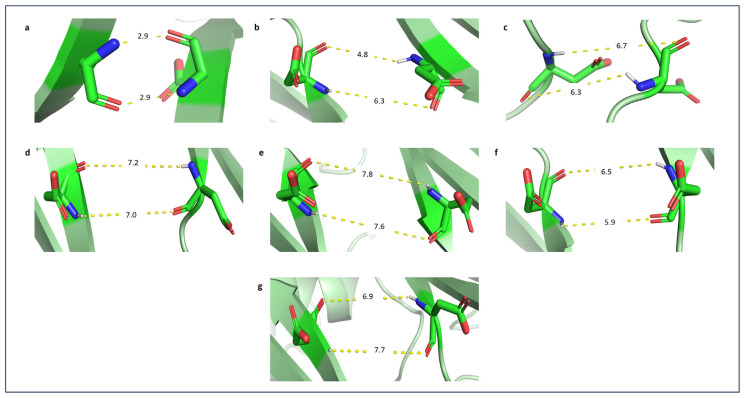
Presence of hydrogen bond between Asp 421-Asp 421 in dimer a) wild type and variant structures b) G6PD_Zacatecas_ (R257L), c) G6PD_Durham_ (K238R), d) G6PD_Valladolid_ (R136C), e) G6PD_Mediterranean_ (S188F), f) G6PD_MexicoCity_ (R227Q), and g) G6PD_A+_ (N126D).

The βN strands of two monomers form the dimer interface. This interface is crucial for enzyme stability and activity since the G6PD enzyme is in dimer–tetramer equilibrium [3,12]. To determine the existence of two hydrogen bonds between Asp421Asp421, which connecting the two βN strands, the computation of the distance between residues was performed. Two hydrogen bonds were present between Asp421-Asp421 at 100ns in the wild type. However, other variants are unable to retain hydrogen bonds between Asp 421 as the distance between the residues is more than 3.3 Å, as indicated in Fig. 10.

There are four salt bridges present at the dimer interface. The establishment of contacts between two subunits of a G6PD dimer is accomplished through salt bridges between highly conserved residues Glu 206–Lys 407 and Glu 419–Arg 427. Table 3 shows that the only variant without the two salt bridges between Glu 206 and Lys 407 is G6PD_Zacatecas_ (R257L). The reduced affinity of structural NADP^+^ in this variant affects the stability of the dimer interface by inducing the loss of the salt bridge. However, the two salt bridges between Glu 419 and Arg 427 were absent in the wild type and all the variants.

### 3.4. Affinity of ligands

**Fig. 11:**
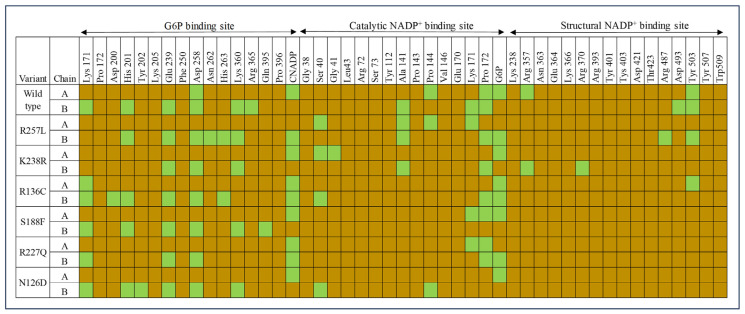
Heatmap illustrating the ligand binding pockets’ occupancy indicated by hydrogen bond presence (green) and absence (brown).

The study compared the enzyme function of G6PD wild type and variants, analyzing the impact of molecular changes. The occupancy of ligand binding pockets compared with the enzyme affinity values derived from biochemical data of K_m_ G6P and K_m_ catalytic NADP ^+^ [10,39]. The enzyme’s affinity for G6P and catalytic NADP^+^ varies across each variant, with different K_m_ values listed in Table 4. Higher K_m_ values indicate lower protein-ligand affinity based on biochemical data [40]. As per the heatmap of ligand binding pockets occupancy, it is evident that the wild type structure in chain A exhibits a lower affinity for G6P. However, it is capable of forming robust hydrogen bonds with catalytic NADP^+^. Chain B shows higher G6P affinity and is able to retain Lys 171, which is crucial for oxidation and catalysis of the G6P substrate. The catalytic NADP^+^ has a lower affinity in chain A and a higher affinity in chain B due to a hydrogen bond with Lys 171, as shown in Fig. 11. The wild type also showed significant occupancy at the structural NADP^+^ binding site, which accounts for its robust structural integrity at the dimer.

G6PD_Durham_ (K238R), G6PD_Valladolid_ (R136C), G6PD_Mexicocity_, and G6PD_A+_ show lower affinity towards G6P chain A and higher affinity for chain B which is similar to wild type. However, G6PD_Zacatecas (R257L) and G6PDMediterrenean_ (S188F) lost affinity towards G6P on chain A and lower affinity at chain B. Results of G6PD_Zacatecas_ (R257L) correlate with higher G6P K_m_ value reported in biochemical data.

Moreover, G6PD_Durham_ (K238R), G6PD_Zacatecas_ (R257L), G6PD_Valladolid_ (R136C), and G6PD_Mexicocity_ (R227Q) show similar ligand affinity as wild type for catalytic NADP^+^. G6PD_Mediterrenean_ (S188F) lost its affinity for catalytic NADP^+^ in chain B but exhibited higher affinity in chain A. The affinity of both chains of G6PD_A+_ (N126D) for catalytic NADP^+^ is notably lower. This correlation is consistent with the reported catalytic NADP^+^ K_m_ results. G6PD_Zacatecas_ (R257L), G6PD_Durham_ (K238R), G6PD_Valladolid_ (R136C) have lower affinity towards structural NADP^+^. While G6PD_Mediterrenean_ (S188F), G6PD_Mexicocity_ (R227Q), and G6PD_A+_ (N126D) lost affinity for structural NADP^+^.βE–αe loop serves a vital role in G6PD catalysis with the presence of critical residues Lys 171 involved in substrate oxidation and catalysis (3). From the results reported in Fig. 11 G6PD_Zacatecas_ (R257L) and G6PD_Durham_ (K238R) are unable to retain G6P-Lys 171 hydrogen bond. While the catalytic NADP^+^ - Lys 171 bond absent in G6PD_Durham_ (K238R), G6PD_Valladolid_ (R136C) and G6PD_A+_ (N126D). G6PD_Zacatecas_ (R257L) and G6PD_Durham_ (K238R) revealed loss of Lys 171 hydrogen bond either with G6P or catalytic NADP^+^. Additionally, the results showed that G6PD variant structures with inadequate structural integrity at dimer interfaces exhibit high enzyme activity. The unstable dimer interface for G6PD_Zacatecas_ (R257L), which is revealed via loss of salt bridges and Asp421-Asp421 hydrogen bond, could be the reason for unable to retain both Lys 171-G6P and Lys 171- catalytic NADP^+^ hydrogen bond via the βE–αe loop. This could be the root cause of their diminished enzyme activity. Furthermore, the loss of hydrogen bond between highly conserved amino acid 238 with structural NADP^+^ and Asp421-Asp421 attributed to the reduced enzyme activity in G6PD_Durham_ (K238R). This is supported by the evidence of loss of Lys 171-G6P and Lys 171- catalytic NADP^+^ hydrogen bonds.

## 4. Conclusion

In summary, a complete dimeric structure with structural NADP^+^, catalytic NADP^+,^ and G6P is crucial to understand protein dimerization which reveal the structural stability and flexibility of the G6PD enzyme. This study stands as the pioneer in simulating a selected G6PD dimer variants with ligands and verifying the results with established biochemical data. The unstable dimer interface is shared among all variants, even though each of them demonstrates unique structural alterations as demonstrated by MD simulations. The dimer interface’s integrity is crucial in determining stable G6PD enzyme activity. Loss of salt bridges and hydrogen bond eventually impact the binding affinity of conserved amino acid residues, which ultimately leads to a significant reduction in G6PD enzyme activity. *In silico* computational techniques can be employed to precisely forecast the stabilization of the dimer interface in chosen G6PD variants in developing new drugs.

## Figures and Tables

**Table 1:** Comparison of the intermolecular interactions between the mutation site and neighbouring residues for the wild type and variants

Mutation Side			Hydrogen Bonds between residues in wild type			Hydrogen Bonds between residues in variants		
		
Position		Mutated Residue	Neighboring residue	Distance (Å)	Bonds	Neighboring residue	Distance (Å)	Bonds
257	αi (255–272)	Arg–Leu	Arg 175	2.9	3	Asn 262	2.8	1
			Phe 253	3.0				
			Asn 262	2.8				
238	α-βG (232–238)	Lys–Arg	Asn 363	2.8	4	Lys 366	2.4	1
			Lys 366 sNADP^+^	3.0; 2.7				
				3.2				
136	βD (136–140)	Arg–Cys	Asn 122	2.2	5	Trp 164	2.2	2
			Gly 131	2.8		Arg 166	1.9	
			Cys 158	3.5				
			Trp 164	2.8				
			Arg 166	3.0				
188	αe (177–190)	Ser–Phe	Ser 184	3.0, 3.1	2	Ser 184	2.2	1
227	αg-βG loop (223–231)	Arg–Gln	Arg 348	2.8	2	Arg 348	1.9	2
			Asp 350	2.8		Asp 350	1.8	
126	αc (115–132)	Asn–Asp	Asn 122	2.8	1	Asn 122	2.1	1

**Table 2:** Average trajectory analysis values for the wild type and variant structures of G6PD dimer.

Structures	Mutation Location	Backbone RMSD (nm)	Cα RMSF atoms (nm)	SASA (nm^2^)	Rg (nm)	Intermolecular HB (Number)
Wild Type	–	0.35	0.18	432	3.71	777
Zacatecas	R257L	0.35	0.17	432	3.71	779
Durham	K238R	0.38	0.18	433	3.70	783
Valladolid	R136C	0.36	0.18	426	3.70	789
Mediterranean	S188F	0.34	0.16	436	3.70	787
Mexico City	R227Q	0.37	0.19	437	3.72	777
A+	N126D	0.38	0.15	435	3.71	790

**Table 3:** Distance of salt bridge residues for G6PD dimer wild type and variant structures.

Structures	Mutation Location	Glu 206 - Lys 407	Glu 419 - Arg 427
Wild Type	–	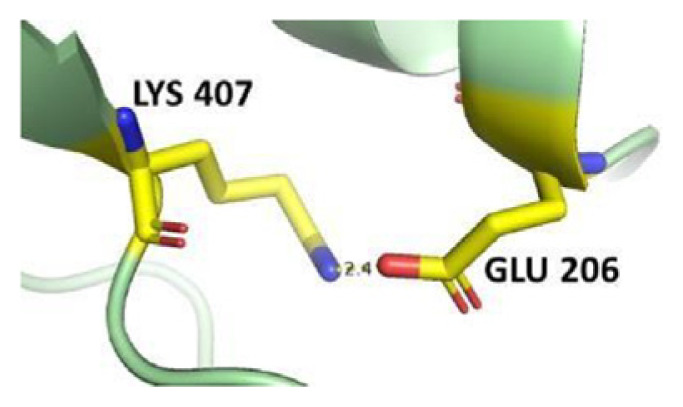	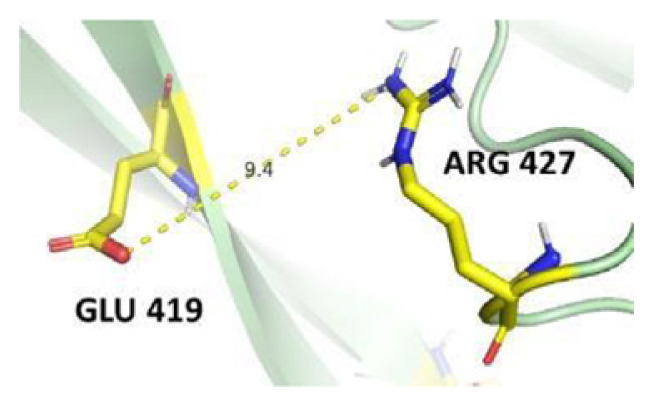
Zacatecas	R257L	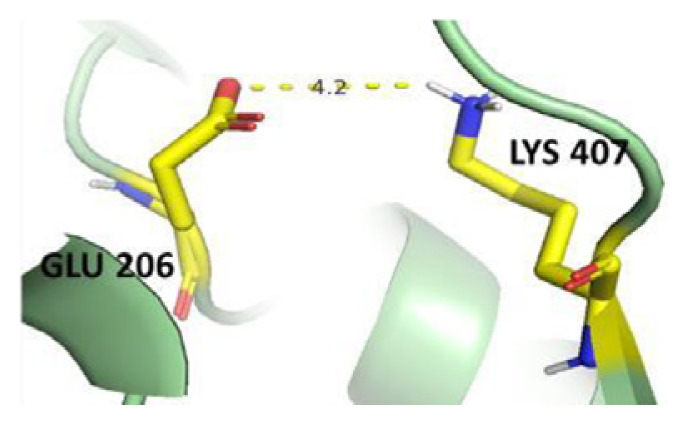	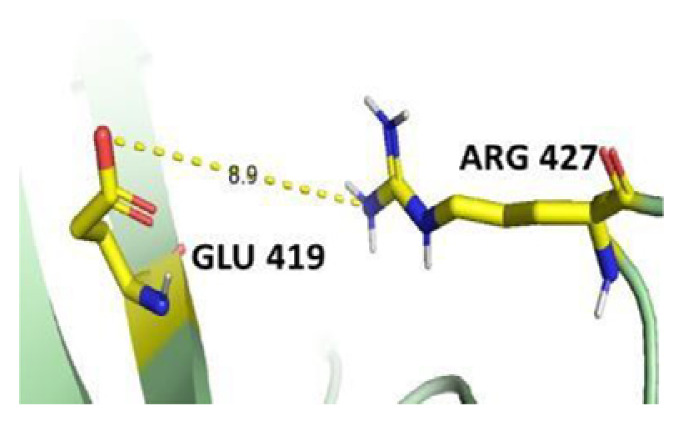
Durham	K238R	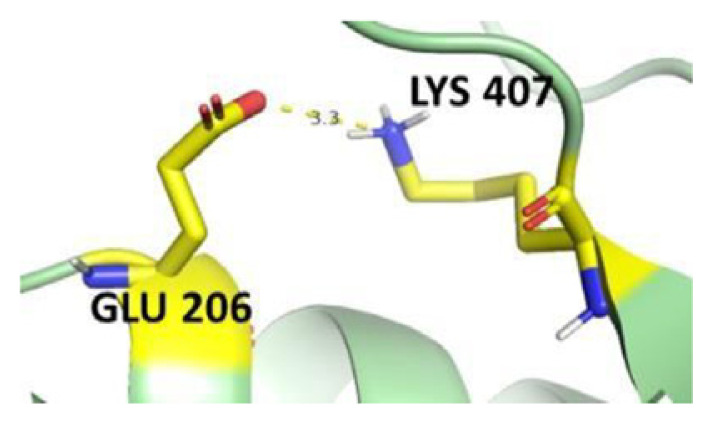	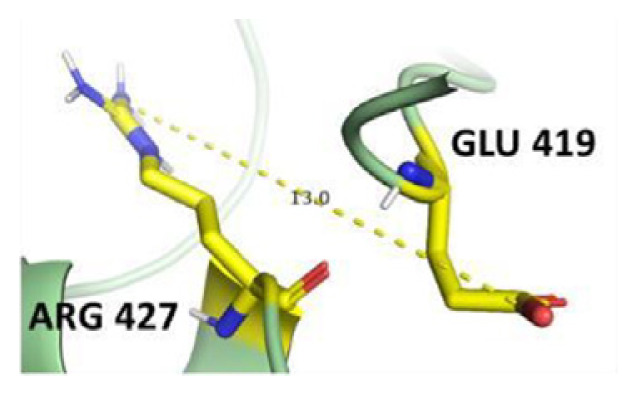
Valladolid	R136C	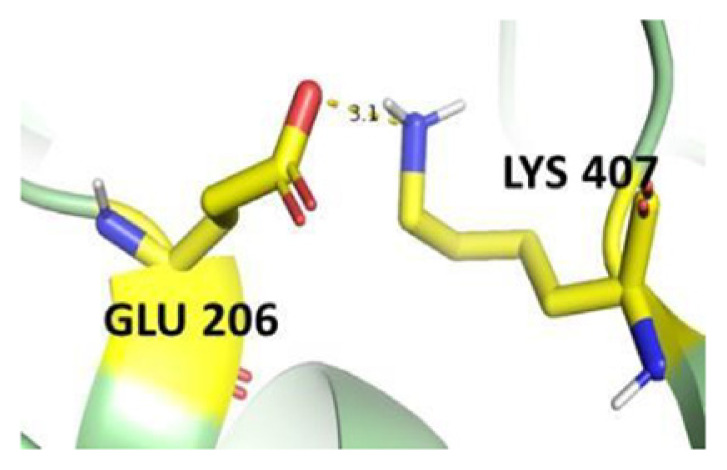	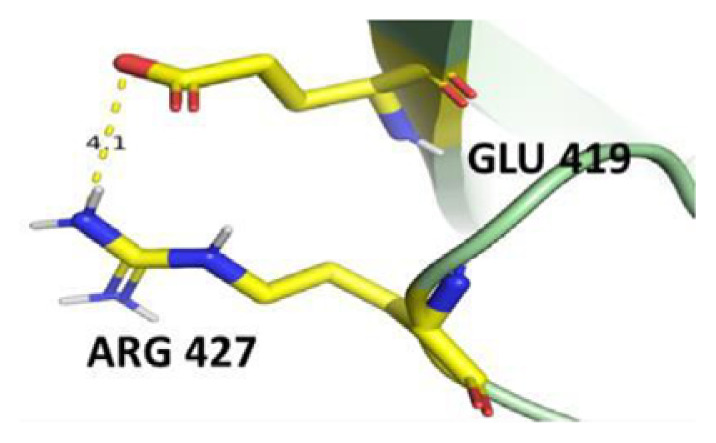
Mediterranean	S188F	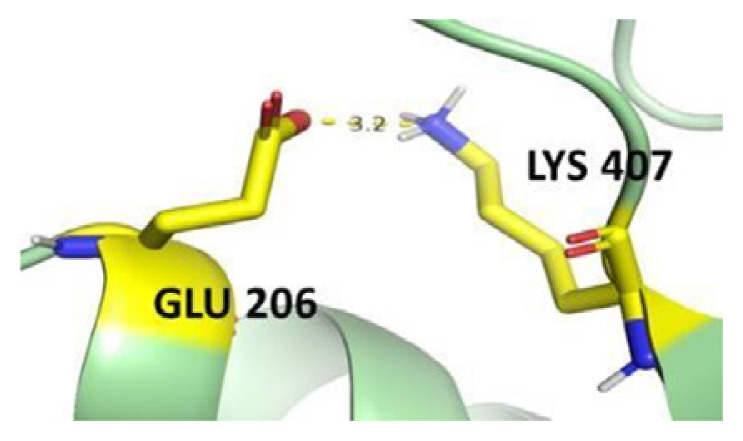	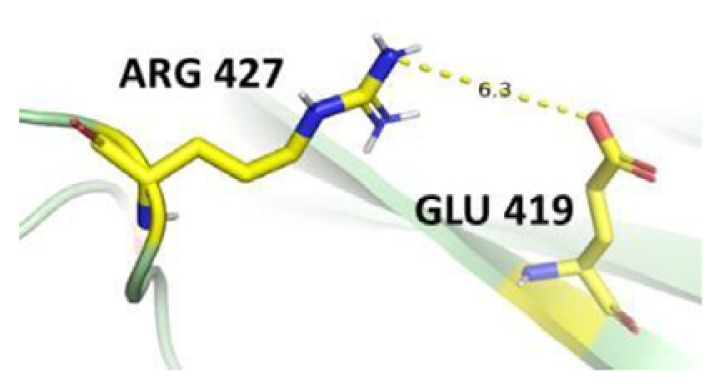
Mexico City	R227Q	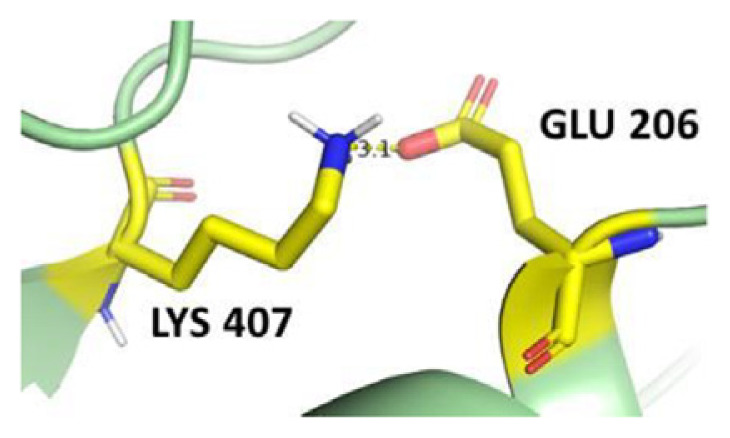	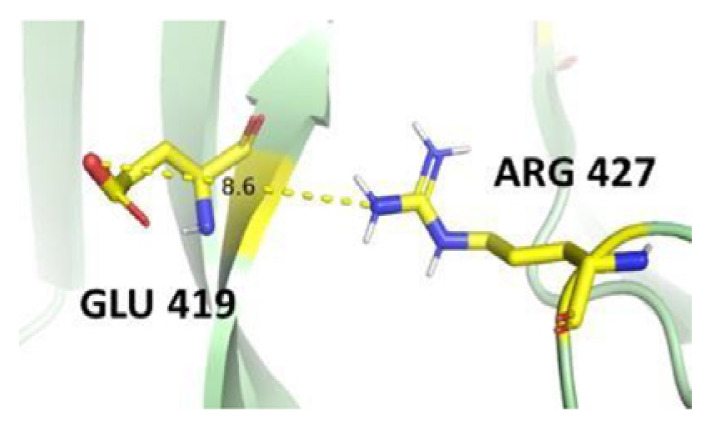
A+	N126D	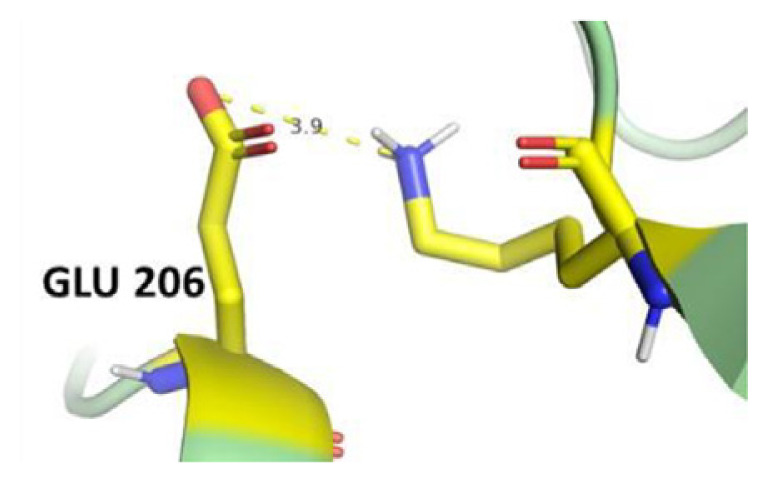	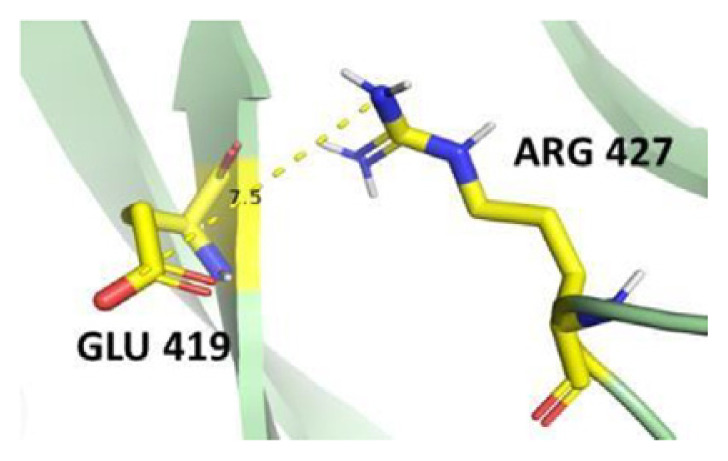

**Table 4:** Summary of steady-state kinetic parameters of human recombinant G6PDs proteins and hydrogen bond results of G6P substrate and catalytic NADP^+^ [10,39].

Variants	Kcat (s-1)	Km G6P (μM)	Km Catalytic NADP^+^ (μM)
Wild Type	233	38.5	6.2
R257L	58	111	24
K238R	71	24.77	7.0
R136C	96	21.5	3.6
S188F	NA	23	1.2–1.6
R227Q	182	24.9	9.1
N126D	114	56.4	12.97
